# Multiple regions in the extracellular domain of the glycine receptor determine receptor activity

**DOI:** 10.1074/jbc.RA118.003088

**Published:** 2018-06-25

**Authors:** Bijun Tang, Sarah C. R. Lummis

**Affiliations:** From the Department of Biochemistry, University of Cambridge, Cambridge CB2 1QW, United Kingdom

**Keywords:** glycine receptor, electrophysiology, membrane biophysics, mutagenesis, mutant, aromatic amino acid, Cys-loop, ion channel, ligand-gated, pentameric ligand-gated ion channel, two-voltage electrode clamp

## Abstract

Glycine receptors (GlyRs) are Cys-loop receptors that mediate fast synaptic inhibition in the brain stem and spinal cord. They are involved in the generation of motor rhythm, reflex circuit coordination, and sensory signal processing and therefore represent targets for therapeutic interventions. The extracellular domains (ECDs) of Cys-loop receptors typically contain many aromatic amino acids, but only those in the receptor binding pocket have been extensively studied. Here, we show that many Phe residues in the ECD that are not located in the binding pocket are also involved in GlyR function. We examined these Phe residues by creating several GlyR variants, characterizing these variants with the two-electrode voltage clamp technique in *Xenopus* oocytes, and interpreting changes in receptor parameters by using currently available structural information on the open and closed states of the GlyR. Substitution of six of the eight Phe residues in the ECD with Ala resulted in loss of function or significantly increased the EC_50_ and also altered the maximal response to the partial GlyR agonist taurine compared with glycine in those receptor variants that were functional. Substitutions with other amino acids, combined with examination of nearby residues that could potentially interact with these Phe residues, suggested interactions that could be important for GlyR function, and possibly similar interactions could contribute to the function of other members of the Cys-loop receptor family. Overall, our results suggest that many ECD regions are important for GlyR function and that these regions could inform the design of therapeutic agents targeting GlyR activity.

## Introduction

Glycine receptors (GlyRs)^2^ mediate fast synaptic inhibition in the brain stem and spinal cord and are involved in the generation of motor rhythm, coordination of reflex circuits, and processing of a variety of sensory signals, such as pain (1). They are members of the Cys-loop (or pentameric ligand-gated ion channel, pLGIC) receptor family along with nicotinic ACh (nACh), 5-HT_3_, and GABA_A_ receptors (2). In these receptors, five subunits are arranged pseudosymmetrically around a central ion-conducting pore; each subunit comprises a large N-terminal ligand-binding domain and four transmembrane helices that are connected by loops of varying sizes. Four GlyR α subunits and one GlyR β subunit are known to date.

GlyRs have proved to be the vertebrate Cys-loop receptor of choice for high-resolution structural studies, and there are currently a number of published structures bound to a variety of ligands; these provide a reasonable view of different states of the receptor (*e.g.* resting, open, and closed (3–5)). These data support many previous mutagenesis studies, which have previously identified important regions of the protein. They have confirmed, for example, that the orthosteric (agonist) binding site is located at the subunit interface and is formed by three loops from one subunit (A–C) and three β strands from the adjacent subunit (D–F) (6–10). The binding pockets are rich in aromatic residues, and previous studies have shown that a cation–π interaction between Phe-159 in loop B and the positively charged amine on glycine (9) or on the partial agonists β-alanine and taurine (11) makes a substantial contribution to agonist binding, as does a similar interaction in many other Cys-loop receptors, including nACh, 5-HT_3_, MOD-1, and GABA_A_ receptors (12–17).

**Figure 1.**
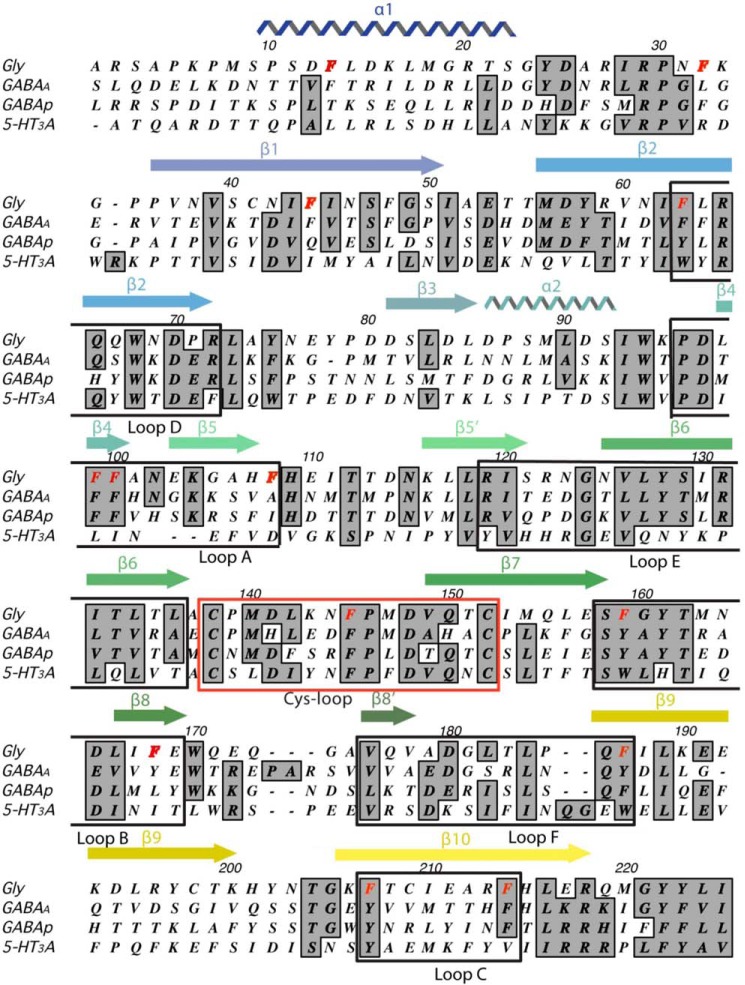
**Clustal alignment of Gly (α1) GABA and 5-HT_3_A receptor subunits showing locations of secondary structural features.** The Phe residues in the GlyR subunit are shown in *red*. Note that most are located in the binding loops.

There are, however, many other Phe residues in the ECD (Fig. 1), and, given that we now have high-resolution structural data, we are in an excellent position to determine what interactions they might form and, when combined with functional data, determine what (if any) roles these residues play in the structure or function of the receptor. This is the aim of this study.

## Results

### WT GlyRs

**Figure 2.**
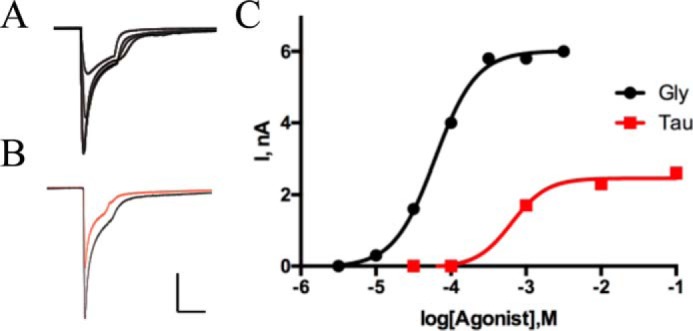
**Responses to glycine and taurine in WT GlyR.** *A*, typical responses to the application of a range of concentrations of glycine to an oocyte expressing WT GlyR. *B*, typical maximal responses to glycine (1 mm) and taurine (*red*; 100 mm) in an oocyte expressing WT GlyR show that taurine is a partial agonist. *Scale bars*, 2 μA, 30 s. *C*, typical concentration–response curves for glycine and taurine in a single oocyte expressing WT GlyR. Data are typical of at least three oocytes.

GlyRs have been previously well-studied using heterologous systems, and parameters we obtained from concentration–response curves following expression in *Xenopus* oocytes (EC_50_ = 49 μm, *n*_H_ = 2.5) are consistent with previous studies (*e.g.* see Pless *et al.* (11)). In addition to examining responses with glycine, we also determine the maximal responses obtained with the partial agonists β-alanine and taurine; we observed that β-alanine was close to a full agonist (80 ± 8%), whereas taurine yielded maximal responses of 51 ± 7% (Fig. 2).

### Phe residues in or near the N-terminal α-helix

**Figure 3.**
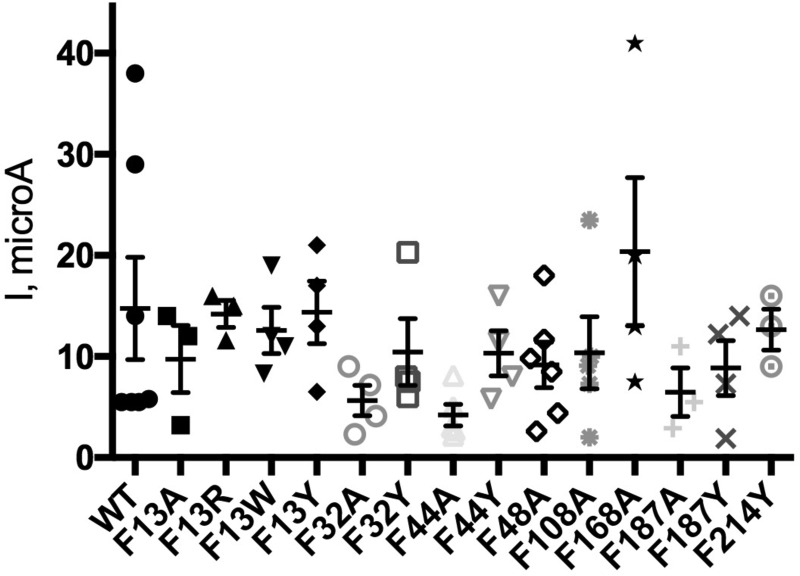
**Scatter plot showing maximal responses of WT and mutant GlyR.** Bars show mean ± S.E., *n* = 3–8. No values are significantly different from WT (ANOVA with Dunnett's post hoc test).

There are two Phe residues in or near the N-terminal α-helix; Phe-13 and Phe-32. Substitution of either of these with Ala resulted in large changes in the glycine EC_50_, indicating that they are important for the correct function of the receptor (Table 1). Maximal responses from these (and all other mutant receptors that we created) had values that were not significantly different from those for WT receptors, indicating no expression problems (Fig. 3). To probe whether EC_50_ changes might be due to alterations in binding affinity or channel gating, we determined maximal responses with taurine in these mutants. Both had changes to *I*_max_ taurine/*I*_max_ glycine (Table 1); taurine was a full agonist in F13A-containing mutant GlyR with maximal taurine responses of 97% compared with maximal glycine responses, whereas F32A-containing GlyR had significantly lower relative maximal taurine responses (14%) than WT receptors (51%).

**Table 1:** **WT and Phe mutant GlyR parameters** Data are mean ± S.E., *n* = 4–8; *, significantly different from WT, *p* < 0.05 ANOVA with Dunnett's post-comparison test; NR, non-responsive; ND, not determined.

Mutant	pEC_50_	EC_50_	%*I*_max_ Tau/*I*_max_ Gly	*n*_H_
	*m*	μ*m*		
WT	4.31 ± 0.04	49	51 ± 7	2.5 ± 0.3
F13A	2.98 ± 0.06*	1050	97 ± 15*	3.2 ± 0.4
F13W	4.17 ± 0.04	67	70 ± 5	2.0 ± 0.3
F13Y	3.00 ± 0.03*	998	53 ± 11	2.4 ± 0.2
F13R	3.32 ± 0.06*	478	88 ± 6*	2.8 ± 0.4
F13E	3.10 ± 0.07*	796	46 ± 9	2.1 ± 0.5
F32A	2.79 ± 0.08*	1600	14 ± 4*	2.4 ± 0.2
F32Y	3.92 ± 0.04*	120	89 ± 8*	2.2 ± 0.3
F44A	2.20 ± 0.03*	6320	10 ± 3*	2.0 ± 0.2
F44Y	4.21 ± 0.05	62	80 ± 9	2.3 ± 0.2
F48A	3.95 ± 0.02*	112	36 ± 3	
F63A (11)		89,000 ± 6000		
F63Y (11)		2000 ± 400		
F99A (22)		82 ± 28	ND	
F100A (22)		34 ± 14	ND	
F108A	4.28 ± 0.01	52	70 ± 8	2.2 ± 0.4
F145A (21)		775 ± 69	52 ± 3	
F159A (11)		8100 ± 200	86	
F159Y (20)		13.2 ± 1.0	95	
F168A	4.11 ± 0.06	78	67 ± 5	2.6 ± 0.2
F187A	3.35 ± 0.04*	443	16 ± 4*	2.8 ± 0.4
F187Y	4.95 ± 0.05*	11	66 ± 5	1.7 ± 0.3
F207A (11)		NR		
F207Y (11)		161 ± 23	ND	
F214A		NR		
F214Y	4.15 ± 0.03	70	63 ± 5	2.7 ± 0.2

**Figure 4.**
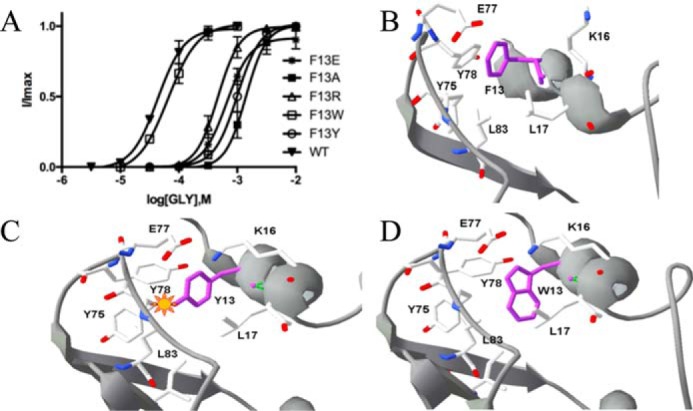
**Phe-13 substitutions.** *A*, concentration–response curves of Phe-13 mutant GlyR show that Phe can be replaced by Trp to give a WT-like curve, but replacement with Tyr, Ala, Glu, or Arg results in rightward shifts. Data = mean ± S.E., *n* = 4–8. Parameters derived from these curves are shown in Table 1. *B*, closed GlyR structure showing residues surrounding Phe-13. Note how far Lys-16 is from Phe-13. *C*, the open GlyR structure shows that Lys-16 is in a different location, and substitution of Phe with Tyr results in a steric clash with Tyr-75, whereas replacement with Trp does not (*D*).

Examination of the structure of the receptor in the region of these residues shows that there are multiple possible interactions that could be made by Phe-13 with other residues in the subunit: a π–π interaction with Tyr-75 or Tyr-78, a cation–π interaction with Lys-16, an anion–π interaction with Glu-77, and hydrophobic interactions with Leu-17 and Leu-83. To explore these potential interactions, we made further substitutions: F13W, F13Y, F13R, F13E, K16A, and E77A. F13W-containing receptors had an EC_50_ value similar to that of WT receptors, whereas there were large increases in EC_50_ values in F13A-, F13Y-, F13R-, and F13E-containing GlyR (Fig. 4). The increase with a F13Y mutation is inconsistent with data from the F13W mutation, which suggests that an alternative aromatic can substitute effectively for Phe but in fact can be readily explained by the structural data; substituting a Tyr for Phe here *in silico* results in a steric clash with Tyr-75 (Fig. 4*C*). The structure also reveals that the distances between Phe-13 and the residues mentioned above differ in the open and closed states of the receptor, with the most significant difference being the distance to Lys-16, suggesting the possibility of a cation–π interaction. However, a K16A substitution resulted in receptors with an EC_50_ similar to WT (pEC_50_ = 3.98 ± 0.05, EC_50_ = 106 μm; data are mean ± S.E., *n* = 4), and the relative orientation of this residue and Phe-13 is not optimal for such an interaction, so we consider it unlikely to occur. Similarly, a E77A substitution resulted in WT-like receptors (pEC_50_ = 4.17 ± 0.06, EC_50_ = 67 μm; data are mean ± S.E., *n* = 6), indicating that there is no anion–π interaction. Data from the Arg and Glu substitutions of Phe-13 also suggest that there are no charge interactions here, as they caused large increases in EC_50_ similar to those resulting from an Ala substitution.

**Figure 5.**
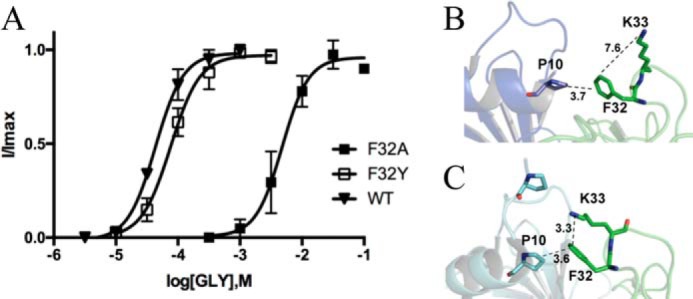
**Phe-32 substitutions.** *A*, concentration–response curves of Phe-32 mutant GlyR show that Phe can be replaced by Tyr to give a WT-like curve, but replacement with Ala results in a rightward shift (WT curve from Fig. 4*A* added for comparison). Data = mean ± S.E., *n* = 4–8. *B*, in the closed GlyR Phe-32 is close to Pro-10 on the adjacent subunit, as it is in the open structure (*C*). However, here it is also close enough to Lys-33 to form a cation–π interaction.

There are fewer possible potential interactions with Phe-32; one of these is a cation–π interaction with Lys-33, which the structure suggests is possible in the open (with a distance of 3.3 Å between these residues) but not the closed state (7.4 Å) of the receptor. We therefore also created and tested receptors containing K33A and K32R substitutions, but these had EC_50_ values similar to WT (K33A: pEC_50_ = 4.23 ± 0.1, EC_50_ = 54 μm; K33R: pEC_50_ = 4.46 ± 0.02, EC_50_ = 35 μm; data are mean ± S.E., *n* = 4). Another possible interaction of Phe-32 is with the adjacent subunit, where it could interact with Pro-10 (Fig. 5). A Pro–Phe interaction may seem unlikely, but Pro interacts well with aromatic residues due to its polarized C–H bond interacting with the π face (a CH–π interaction) (18). However, a P10A substitution resulted in no change in potency (pEC_50_ = 4.12 ± 0.02, EC_50_ = 76 μm; data are mean ± S.E., *n* = 4). The similar distances between Phe-32 and this Pro in the open and closed states (3.6 and 3.7 Å, respectively) also suggest that if such an interaction exists, it does not play a role in function.

### Binding site Phe residues

**Figure 6.**
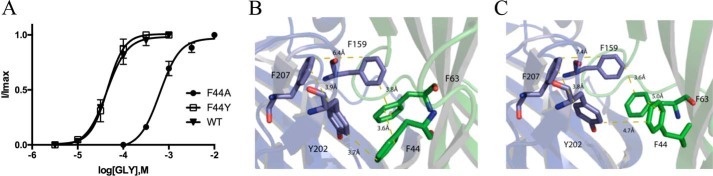
*A*, concentration–response curves of Phe-44 mutant GlyR show that Phe can be replaced by Tyr to give a WT-like curve, but replacement with Ala results in a rightward shift (WT curve from Fig. 4*A* added for comparison). Data = mean ± S.E., *n* = 4–8. *B* and *C*, aromatic residues in the GlyR binding pocket in the open (*B*) and closed (*C*) states.

The Phe residues that are located in the binding pocket (Phe-63 (loop D), Phe-99 (loop A), Phe-100 (loop A), Phe-159 (loop B), and Phe-207 (loop C)) have been investigated previously (9, 19–22); the data from substitutions to these residues are included in Table 1 for completeness. However, Phe-44, Phe-108, and Phe-168, which are located behind the binding pocket, have not been studied previously. Our data show that Ala substitutions to Phe-44 resulted in the largest change in EC_50_ compared with WT receptors that we observed for any of our mutant receptors (Table 1) and also caused the largest reduction in the maximal taurine response. This residue is located below Tyr-202 and Phe-63 and is in a position to form a π–π interaction with either of these residues in the open state (Fig. 6). Further exploration of Phe-44 by substitution with Tyr revealed WT-like properties, confirming the importance of the aromatic ring. We note that Phe-63, which also causes a large increase in EC_50_ when mutated to Ala (7, 11), could also form a π–π interaction with Phe-159, a residue that has long been known to be important for agonist binding (19), and therefore an interaction with Phe-44 may not occur. Thus, we propose that Phe-44 probably interacts with Tyr-202.

**Figure 7.**
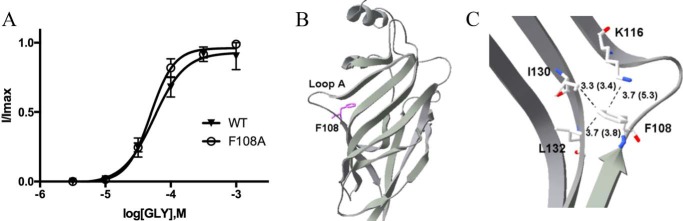
*A*, concentration–response curves of Phe-108 mutant GlyR show that Phe can be replaced by Ala to give a WT-like curve. Data = mean ± S.E., *n* = 4–8. *B*, Phe-108 is located at the end of loop A, where it could have hydrophobic interactions with Ile-130 and Leu-132 (*C*) and perhaps a cation–π interaction with Lys-116. Distances between residues in the open (and closed) states are shown.

The EC_50_ value for F108A-containing GlyR (52 μm) is similar to WT, and the mutation did not cause a change in the maximal response to taurine (Table 1). This residue is located 3.7 Å from Lys-116 in the closed state, giving it the potential to form a cation–π interaction here, with this distance increased to 5.3 Å in the open state (Fig. 7). However, the functional data suggest no effect of removing the aromatic, so we propose that such an interaction does not occur. The data are nevertheless not inconsistent with this Phe contributing to a hydrophobic region that includes Ile-130 and Leu-132.

**Figure 8.**
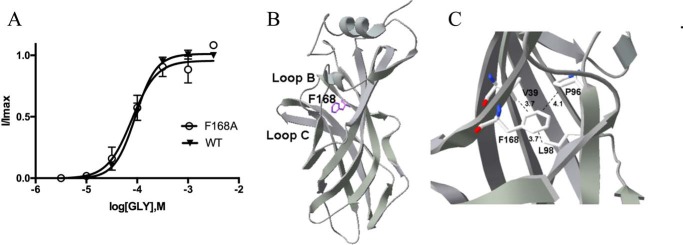
*A*, concentration–response curves of Phe-168 mutant GlyR show that Phe can be replaced by Ala to give a WT-like curve. Data = mean ± S.E., *n* = 4–8. *B* and *C*, Phe-168 is located at the end of loop A, where it could have hydrophobic interactions with Val-39, Leu-98, and Pro-96 and/or a Pro–π interaction with Pro-96. Distances between residues in the open and closed states are similar.

The EC_50_ value for F168A-containing GlyR (78 μm) is also similar to that of WT receptors, and again this substitution did not cause a change in the maximal response to taurine (Table 1). This residue is located at the end of loop B, and, although the structure shows that it has the potential to form a Pro–π interaction with Pro-96 (Fig. 8), the data do not support such an interaction, and we suggest that Phe-168 only contributes to hydrophobic interactions here.

### Phe residues at the ECD–TMD interface

**Figure 9.**
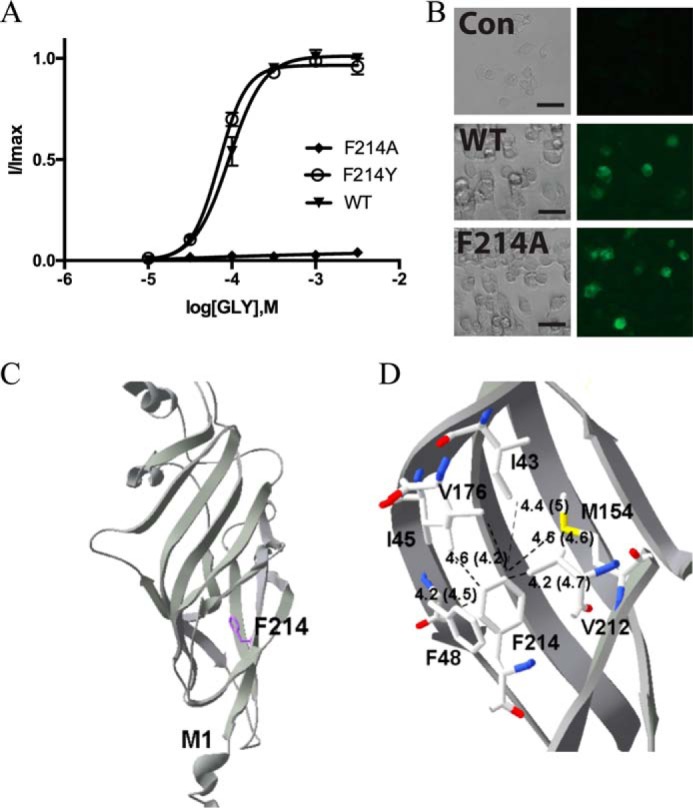
*A*, concentration–response curves of Phe-214 mutant GlyR show that Phe can be replaced by Tyr to give a WT-like curve, but replacement with Ala ablated the response to glycine (WT curve from Fig. 8*A* added for comparison). Data = mean ± S.E., *n* = 4–8. *B*, probing with GlyR antisera reveals that F214A-containing GlyR are expressed in HEK293 cells. *Con*, untransfected cells. *Scale bar*, 30 μm. Results are typical of three experiments. *C*, Phe-214 faces into the subunit and could have multiple hydrophobic interactions, which are shown in *D*. There is also the possibility of a π–sulfur interaction with Met-154. Distances between residues in the open (and closed) states are shown.

The largest number of Phe substitutions we probed were at or close to the ECD–TMD interface, and all Ala mutations caused changes in EC_50_ values and/or maximal taurine responses. F48A-containing GlyR had a 2-fold increase in EC_50_ compared with WT, with no change in the relative taurine response. This is interesting, as the structure reveals that Phe-48 on the β1 strand could form a π–π interaction with Phe-214, which is on the β10 strand, which links to M1. In addition, Phe-48 could interact with Tyr-58, which is located close to the binding pocket. However, the relatively small change in EC_50_ with F48A suggests that the Phe here does not play an especially critical role in either binding or function. This is in contrast to Phe-214, which is sensitive to removal of the aromatic group; F214A-containing GlyR were nonfunctional despite being expressed (Fig. 9). Phe-214 is located in a part of the subunit where it could interact with many hydrophobic residues; substitution with Ala would mean it was too distant to allow most of these interactions. The structural data show there is also the potential for a π–sulfur interaction with the sulfur on Met-154. However, the distance between Phe-214 and Met-154 does not differ between open and closed states, and the residues are too far apart for optimal interaction (<4.3 Å) (23), so we consider such an interaction unlikely, and if it does occur, it is probably not involved in receptor activation.

**Figure 10.**
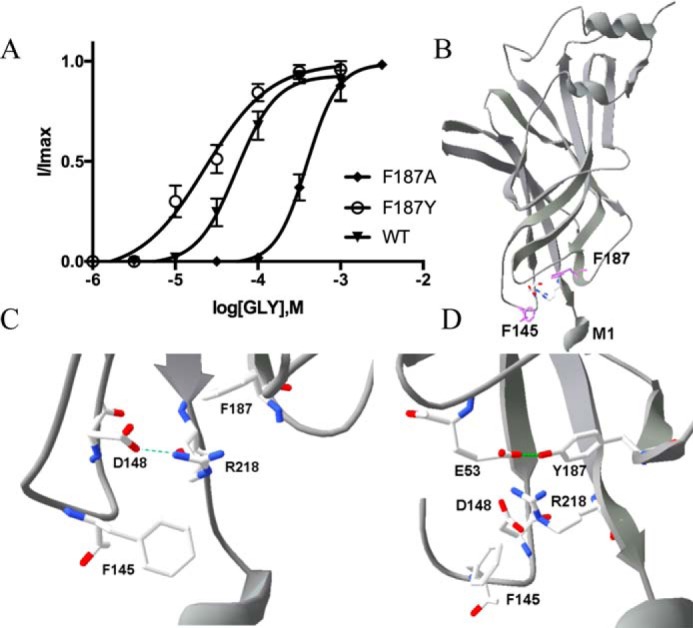
*A*, concentration–response curves of Phe-187 mutant GlyR show that replacement with Ala shifts the curve to the *right*, whereas replacement with Tyr shifts the curve to the *left* (WT curve from Fig. 7*A* added for comparison). Data = mean ± S.E., *n* = 4–8. *B* and *C*, Phe-145 and Phe-187 are located at the TMD–ECD interface on either side of the salt bridge formed by Asp-148 and Arg-218. *D*, substitution of Phe-187 with Tyr reveals a possible hydrogen bond with Glu-53.

Phe-145 and Phe-187 have been studied previously (10, 20). Data indicate that they provide a hydrophobic environment for an important salt bridge between Asp-148 and Arg-218. This hypothesis would explain our novel data from F187A-containing GlyR (∼10-fold increase in EC_50_), and the structure provides an explanation for the gain of function we observed with F187Y-containing GlyR, a possible hydrogen bond with Glu-53 that could stabilize the structure (Fig. 10).

## Discussion

Here, we show that a number of Phe residues in the ECD of the glycine receptor, which have not been previously identified as important, play a role in the function of the receptor. Some of these are located at or close to the ECD–TMD interface or close to the orthosteric binding site and thus could reasonably be expected to maybe affect ligand binding or receptor activation, but we also identify a novel region in the N terminus of the receptor. This region and the other Phe residues we identified as important are discussed in more detail below.

### Phe residues in or near the N-terminal α-helix

The large increases in EC_50_ caused by Ala substitutions of the two Phe residues in or close to the N terminus were unexpected, as these residues are some distance from the binding pocket and are not on the pathway between the binding site and the transmembrane domain and so would not be predicted to be an essential part of the conformational change needed to transduce agonist binding to channel opening. Nevertheless, changes in taurine efficacy are consistent with the latter (*i.e.* suggest that the mutations effect receptor gating). The N-terminal portion of Cys-loop receptors in the nACh receptor incorporates the main immunogenic region (MIR), which comprises the MIR loop (residues 60–81 in the α1 subunit) and the N-terminal α-helical region (residues 2–14). The MIR is the binding site for many antibodies that cause myasthenia gravis, an autoimmune disease targeting the neuromuscular junction of skeletal muscle (24). Such antibodies block the ACh-induced response, but the mechanism by which they achieve this may not be by a physical occlusion of the pore or binding pocket, as originally proposed, but instead may involve inhibition of the conformational change required for channel opening. Such a mechanism was proposed by Lou *et al.* (25) based on their data showing large changes in sensitivity to ACh following alterations to the MIR, combined with molecular dynamics simulation data from Henchman *et al.* (26) showing conformational changes in this region associated with receptor activation. Our data are consistent with an important functional role in this region of the protein, which may be essential for efficient channel gating. This may involve interactions between the N-terminal α-helix and the β1–β2 loop (which is located between loops D and A and thus could be directly influenced by agonist binding) and also between the N-terminal α-helix and the adjacent subunit. We propose these interactions are mediated at least in part via Phe residues in the GlyR. Similar interactions may occur in other Cys-loop receptors, but given the different lengths of the N-terminal α-helix and the regions between loops A and D in different receptors, the specific residues mediating these interactions may not be conserved.

In support of this hypothesis, we note that the GlyR positive allosteric modulator AM-3607 binds in this region (5). This compound, which increases glycine-binding affinity and potentiates channel activity, has been shown to bind to what has been designated a novel allosteric site. Binding of positive allosteric modulators to this site has not been observed previously for any Cys-loop receptor; therefore, these data, combined with our and other mutagenesis studies, suggest an exciting new potential therapeutic binding site not only for GlyR but also possibly other pLGICs.

### Phe residues in the GlyR-binding site

Aromatic residues in the binding site (Phe-63, Phe-159, Tyr-202, and Tyr-207) form the “aromatic box,” which is a well-established feature of all Cys-loop receptors, and these residues in the GlyR have been extensively investigated by several groups. In particular, data have shown the importance of Phe-159, which forms a critical cation–π interaction with the natural agonist (9) and also with the partial agonists β-alanine and taurine (11). In the current study, we show that Phe-44, which is located just behind the binding pocket close to Tyr-202, also plays a role in receptor function and suggest that this is due to the formation of a π–π interaction with Tyr-202 (*i.e.* in effect tethering this aromatic box residue). Tyr-202 is in loop C, a region that undergoes significant change in conformation following agonist binding (loop C capping) and is likely the first step in the series of conformational changes that ultimately result in channel opening in Cys-loop receptors (3, 27). Thus, incorrect positioning of Tyr-202 could have a significant effect on this event, which would be consistent with our taurine data that indicate a role of this residue in the gating process.

Not all Phe residues located near the binding pocket, however, alter receptor function; our data from F108A- and F168A-containing Gly receptors revealed that aromatic residues are not essential at these locations.

### Phe residues at or close to the ECD–TMD interface

The interface between the ECD and the TMD is a critical region for transducing agonist binding to channel gating. The most detailed work on this area has combined structural and functional studies in ELIC and GLIC (28), and this work, combined with data from many other studies, has shown that there is unlikely to be a range of conserved sets of critical pairwise ECD–TMD bonds, although it is clear that charged residue interactions are especially important (29–32). Aromatic residues can be involved in such interactions via their π rings, but they also can play other roles. A particular case is that of Phe-145 and Phe-187 in the GlyR, which have been extensively investigated by Pless *et al.* (10). These authors concluded that these residues provide a hydrophobic framework for a strong electrostatic interaction between Asp-148 in the Cys-loop and Arg-218 in M1. As altering either of these residues has a major effect on EC_50_, it is likely this salt bridge is critical for function. Pless *et al.* (10) suggest that the interaction is present in both open and closed receptors, but as the distances between these residues decrease from the closed (3.3 Å) to the open (2.6 Å) states (Fig. 10), we propose that this salt bridge may form as part of the activation process. This hypothesis is supported by the changes in taurine efficacy observed with Ala substitutions of these and the two Phe residues, which are also consistent with a role in gating.

Our data also indicate that an aromatic residue is critical at position 214, but not at position 48, ruling out a critical role for the π–π interaction between these two residues, which is suggested by the structural data (Fig. 9). We propose instead that hydrophobic interactions of Phe-214 are important in this region for the correct functioning of the receptor; Phe-214 is not only close to the interface region between the TMD and the ECD, but it is also part of loop C and can interact with residues in loop A (Ile-43 and Ile-45, which are adjacent to Phe-44, whose importance is discussed above) and loop F (Val-176). The distances of these residues from Phe-214 vary in the open and closed states of the receptor (Fig. 9), supporting our hypothesis that this region, coordinated via Phe-214, is critical for linking agonist binding with channel opening.

Thus, in conclusion, we have shown that many Phe residues located in a range of regions throughout the ECD are involved in the binding and/or function of GlyRs. Given that Cys-loop receptors, such as the GlyR, are targets for a range of therapeutic agents, these data identify new regions that could be used for the design of novel drugs, and they also provide an explanation for the actions of modulators, such as AM-3706, which enhance GlyR function.

## Experimental procedures

### Oocyte maintenance

*Xenopus laevis* oocyte-positive females were purchased from NASCO (Fort Atkinson, WI) and maintained according to standard methods. Harvested stage V-VI *Xenopus* oocytes were washed in four changes of Ca^2+^-free ND96 (96 mm NaCl, 2 mm KCl, 1 mm MgCl_2_, 5 mm HEPES, pH 7.5), de-folliculated in 1.5 mg ml^−1^ collagenase Type 1A for ∼2 h, washed again in four changes of ND96 (as above plus 1.8 mm CaCl_2_), and stored in ND96 containing 2.5 mm sodium pyruvate, 50 mm gentamycin, 0.7 mm theophylline.

### Human embryonic kidney 293 (HEK293) cell culture

HEK293 cells were maintained on 90-mm tissue culture plates at 37 °C and 7% CO_2_ in a humidified atmosphere. They were cultured in Dulbecco's modified Eagle's medium/nutrient mix F-12 (1:1) with GlutaMAX^TM^ I medium (Invitrogen, Paisley, UK) containing 10% fetal calf serum. For immunofluorescent studies, cells on cover slips were transfected using polyethyleneimine. 30 μl of polyethyleneimine (1 mg/ml), 5 μl of cDNA, and 1 ml of Dulbecco's modified Eagle's medium were incubated for 10 min at room temperature, added dropwise to an 80–90% confluent plate, and incubated for 2–3 days before use.

### Receptor expression

cDNA was cloned into pGEMHE for oocyte expression and pcDNA3.1 (Invitrogen) for expression in HEK293 cells. Mutagenesis was performed using QuikChange (Agilent Technologies Inc.). cRNA was *in vitro* transcribed from linearized pGEMHE cDNA template using the mMessage mMachine T7 transcription kit (Ambion, Austin, TX). Oocytes were injected with 50 nl of ∼400 ng μl^−1^ cRNA, and currents were recorded 1–4 days postinjection.

### Electrophysiology

Using a Robocyte voltage-clamp system (Multi Channel Systems, Reutlingen, Germany), *Xenopus* oocytes were clamped at −60 mV. Currents were recorded at a frequency of 5 kHz and filtered at 1 kHz. Micro-electrodes were filled with 3 m KCl. Pipette resistances ranged from 1.0 to 2.0 megaohms. Oocytes were perfused with saline at a constant rate of 1 ml min^−1^. Drug application was via a simple gravity-fed system calibrated to run at the same rate. Extracellular saline contained 96 mm NaCl, 2 mm KCl, 1.8 mm CaCl_2_, 1 mm MgCl_2_, and 5 mm HEPES, pH 7.4, with NaOH.

Analysis and curve fitting were performed using Prism (GraphPad Software, Inc., La Jolla, CA). Concentration–response data for each oocyte were normalized to the maximum current for that oocyte. Statistical significance was determined using an ANOVA with a Dunnett's multiple-comparison post hoc test; *p* < 0.05 was taken as statistically significant.

### Immunofluorescence

This was as described previously (33). Briefly, transiently transfected HEK293 cells were fixed (4% paraformaldehyde), washed in TBS (0.1 m Tris, pH 7.4, 0.9% NaCl), and incubated overnight at 4 °C in anti-glycine receptor α1 C-15 antisera (Santa Cruz Biotechnology, Inc.) at 1:200. Following further washing, biotinylated anti-goat IgG (Vector Laboratories) and FITC avidin D (Vector Laboratories) were used to detect bound antibody. Coverslips were mounted in Vectashield mounting medium (Vector Laboratories). Immunofluorescence was observed using a Leica fluorescent microscope.

### Structures

GlyR structures 5VDH (open) and 5CFB (closed) were downloaded from the Protein Data Bank and viewed using PyMOL or Swiss-PDBViewer. The GlyR structures are those of the homomeric α3 GlyR, but the majority of residues, and in particular all of the aromatic residues studied here, are identical to those in the α1 GlyR.

## Author contributions

B. T. and S. C. R. L. data curation; B. T. and S. C. R. L. formal analysis; B. T. and S. C. R. L. investigation; S. C. R. L. conceptualization; S. C. R. L. supervision; S. C. R. L. funding acquisition; S. C. R. L. writing-original draft; S. C. R. L. project administration; S. C. R. L. writing-review and editing.

## Footnotes

2 The abbreviations used are:

GlyR: glycine receptor
pLGIC: pentameric ligand-gated ion channel
ACh: acetylcholine
nACh: nicotinic ACh
5-HT_3_: 5-hydroxytryptamine
ECD: extracellular domain
TMD: transmembrane domain
MIR: main immunogenic region
HEK: human embryonic kidney
ANOVA: analysis of variance.
